# Individual integrity and public morality in scientific publishing

**DOI:** 10.1590/1980-5764-DN-2022-V001

**Published:** 2022-05-13

**Authors:** Sergio Della-Sala

**Affiliations:** 1 University of Edinburgh, Human Cognitive Neuroscience, Psychology, Edinburgh, UK.

**Keywords:** Open Access Publishing, Predatory Publishers, Plan-S, Integrity, Scientific Publishing, Publicação de Acesso Aberto, Editores Predatórios, Plano-S, Integridade, Publicação Científica

## Abstract

Science and science reporting are under threat. Knowingly or not, researchers and clinicians are part of this debacle. This is not due so much to the notorious replication crisis, as to our acceptance of lowering common morality for personal gains, including the widespread, deprecable phenomenon of predatory publishing. Rather than fiercefully countering this loathsome practice, academics are accepting, often supporting a masquerade solution: paying several thousand dollars to publish for all their own papers. This new policy will create a disparity across richer and poorer disciplines; will result in concentrating even more in the hands of large, rich, Western institutions, also penalising younger researchers; will kill observational studies and exploratory research; and will make disseminating science depending more on finances than on quality. This article calls for the full awareness of the academic community on the risks of the current situation in scientific publishing.

## RESUMO.

A ciência e os relatórios científicos estão ameaçados. Conscientemente ou não, pesquisadores e médicos fazem parte desse desastre. Isso não se deve tanto à notória crise de replicação, mas à nossa aceitação de rebaixar a moralidade comum para ganhos pessoais, incluindo o fenômeno generalizado e depreciável da publicação predatória. Em vez de combater ferozmente essa prática repugnante, os acadêmicos estão aceitando, muitas vezes até apoiando uma solução de disfarce: pagar vários milhares de dólares para publicar seus próprios artigos. Essa nova política criará uma disparidade entre as disciplinas mais ricas e mais pobres, resultará na concentração ainda maior nas mãos de grandes e ricas instituições ocidentais, penalizando também os pesquisadores mais jovens; matará os estudos observacionais e a pesquisa exploratória e fará com que a divulgação científica dependa mais das finanças do que da qualidade. Este artigo apela à plena consciência da comunidade acadêmica sobre os riscos da situação atual da publicação científica.

## INTRODUCTION

Science and science reporting are under threat. Knowingly or not, researchers and clinicians are part of this debacle. This is not due so much to the notorious replication crisis^1^, as to our acceptance of lowering common morality for personal gains^2^. This article aimed at urging our community to raise its morality bar to rescue itself from the abyss of ridicule towards which we are heading at full speed. I first list behaviours that we should all avoid or abide with, and then I discuss in more detail the current situation in publishing, which calls for the full awareness of the academic community.

## MANUSCRIPT ETIQUETTE

### Aim at good science not at “good results”

Chris Chambers, describing the current methodological sins hampering the thoroughness of scientific publications, laid out his forthright manifesto on how to avoid the pitfalls of favouring “good results” over good science. The most frequent of such drawbacks are summarised in Table 1; see also the guidance offered by the Committee on Publication Ethics (COPE), the International Committee of Medical Journals Editor (ICMJE), the NIH Office of Research Integrity (ORI), the Guidelines for Responsible Conduct Regarding Scientific Communication of the Society for Neuroscience (SfN) or the Publication Practices & Responsible Authorship by the American Psychological Association (APA).

**Table 1.** Common unwise practice that should be avoided in reporting scientific data.

Bad practice/misconduct	Description
p-hacking	Fishing for statistically significant results, massaging the data, cherry-picking them, or adding unplanned participants or data points to rich statistical significance^4^
HARKing	Hypothesising after results are known. Writing the introduction and spelling out the study predictions after the data have been collected
Low statistical power	Not collecting enough data, or dispersing them in salami publications, favouring quantity of papers over their quality
Ignoring the effects of different analyses	Not being aware that little differences in scoring, pre-processing, analysing the data result in large conclusions differences^5^
Lacking definitions	Assuming common understanding of terms or concepts^6^
Framing study within loose assumptions	Lack of appreciation of the difference between intuitive hunches and a sound path between predictions and outcome^7^
Pushing for novelty	Considering replications as mundane and wanting in intellectual adroitness
Knowingly publishing poor data	Influencing appointments, promotions and workload with quantity, rather than quality^8^
Publication bias	Hiding, rejecting or not attempting to publishing null results or negative findings
Not data sharing	Being secretive about one’s own data due to fear of being caught wrong
Statistical fallacies	Using easy statistics rather than proper statistics
Not retracting	Unearthing errors and not retracting the paper in fear of public shame
Owning findings	Failing to appreciate that once published, findings belong to the community; criticisms are raised to results and not to authors (unless fraudulent). Verifications should be welcome^9^
Plagiarism	Lifting material from available literature without proper citation, including rephrasing, translating from a foreign text and reproducing own material (self-plagiarism)^10^
Misappropriation	Mentioning someone else’s ideas without the appropriate acknowledgement via citation of the original work
Misleading	Exaggerating the reach of the study, e.g. by gilding the titles of the paper, spuriously widening its real claims^8^
Hiding conflicts	Not declaring possible conflicts of interests of authors or sponsors

### Data of published papers should be posted, always

Anyone skimming through the daily list of dubious papers, highlighted by the laudable Retraction Watch, should be alarmed by the sheer volume of blunder and fabrication tarnishing scientific articles. One way to contrast this dangerous drift is to require that all data on which a report is based be made available for scrutiny, re-analyses and criticisms. Authors should honour this golden rule, reviewers should demand to see the data, editors should insist that they be transparent, and publishers should assist their archiving in accessible repositories.

### Honorary authorship should be avoided

Too often, names are added to the list of authors, even if their contribution does not qualify them as authors. An author of a scientific paper is anyone who contributed substantially to the study, by designing it, collecting considerable amount of the data reported, analysing or interpreting them. All authors are accountable for the content of the manuscript they sign. Anybody else associated with the study should be acknowledged for their specific work, but not listed as an author, see, for instance, the recommendation of the ICMJE or the criteria laid out by CRediT (Contributor Roles Taxonomy). In particular, authorship should not be offered as an honorary homage to someone in a position of power, nor should it be used as a bargaining chip to obtain career or other advantages. In short, an author is someone who actively partook to the study, practically or conceptually; hence, for example, offering access to a group of patients does not qualify the clinician as author (although there is some ambiguity as to what it qualifies as “resources” in CRediT). Moreover, if used thoroughly and systematically, CRediT may also provide a mechanism to reveal any unbalanced division of tasks and workload due to gender or other personal demographics of the researchers involved in the study^11^.

### Ethical approval should be detailed

Ethics is relevant. Which ethics body approved the reported study or permitted the report of the observation should always be explicit in the manuscript. Avoid the cliché of simply parroting the mantra phrase, “The study received ethical approval and is conducted according to the Declaration of Helsinki.” Be specific and consider ethics as integral part of the study process^12^, not a bureaucratic hurdle to overcome^13^.

### Dissemination should be responsible

Scientists and clinicians blame journalists for poor science reporting in the media. However, often exaggeration in the news is due to the researchers bragging about their findings in academic press releases^14^. Researchers should publicise their results responsibly, showing their interest without embellishing them or overstating their reach. This becomes particularly relevant when promoting the outcome of an individual study, which has not been vetted by other laboratories or thoroughly replicated. Science should be disseminated only when is based on solid evidence^15^, and the reports should be comprehensible without trying too hard to be smart or sensationa^16^.

### Peer review should be protected

The idea generally held about reviewing is that it would benefit from an overhaul, changing its status from a quasi-hobby to a mandatory duty of each academic. Reviewing (and editorial) time should figure in the workload models of universities, it should be taught formally to early career researchers, and possibly it should be financially rewarding for the individuals or their institutions^17^.

The process of peer reviewing is not perfect, does not prevent despicable errors, and does not impede very bad research from entering the literature^18^. Yet, if carried out conscientiously, it is the best quality control system we have for the scientific literature^19^. The process is as good as we make it. All researchers should do their share in reviewing papers in their field and should do so according to the golden rule that, when wearing the reviewer’s hat, they should behave as they would like others to behave when they are at the receiving end (wearing the author’s hat). Hence, reviewers should offer their feedback reasonably fast^20^ and should use a polite tone, be honest in their appraisal, and clear in their requests^21^.

The scientific community should resist the pressure to shortening reviewing time to deadlines incompatible with thoroughness. In Commencement Address at Harvard, Aleksandr Solzhenitsyn stated that, “Hastiness and superficiality are the psychic diseases of the 20^th^ century, and more than anywhere else this disease is reflected in the press” (1978)^22^. This warning duly applies to the current urgency imposed by serious publishers of carrying out editorial duties fast rather than well. This is imposed to compete with the speed at which low-quality outlets are willing to accept papers for publication, often with no questions asked, provided their fees are paid (see below). Genuine publishers should ring-fence quality instead of entering this deranged marketplace.

Indeed, the publishing arena is now marred with the problem of a deluge of below-par publications in unscrupulous outlets. Let us trace our steps to analyse how we got here.

## SCIENTIFIC PUBLISHING: A WRONG TURN

### Plan S

At the end of 2018, the initiative *cOAlition S*, launched *Plan S* which establishes the principle that academic journals should gradually increase the quota of papers they publish in Open Access (OA) starting at the beginning of 2022. The outcome of this policy is that publishing each single academic paper will be charged several thousand dollars. Individual researchers, agencies funding their work, or the institution where they operate will have to bear such expenses. The reaction of the academic world has not been to fight against this decision, rather individual universities, institutions, learned societies and even individual research groups are trying to navigate the system by establishing bilateral deals with the publishing houses, allowing their affiliated researchers to publish their papers at discounted fees. These deals involve packages including a fixed number of papers that each group will be allowed to publish with a particular publisher at no extra cost. The benefit would be that all published material will be made available to everyone in OA.

However, the new policy will also carry severe consequences: (1) Institutions will not cover the entire costs of publications, part of which will have to be met by individual researchers, creating a disparity across richer and poorer disciplines^23^; (2) Publishing rights will be concentrated even more in the hands of large, rich, Western academic institutions, excluding researchers who carry out their studies in less privileged institutions around the world; (3) Observational studies, single cases, exploratory research, serendipitous findings, or any study not fully funded by granting bodies but also position papers, viewpoints, discussions, and commentaries will be discouraged; (4) Younger researchers with less access to large amounts of financial support for their research will be penalised, forcing them to team up with wealthier colleagues to see their results published^24^; and (5) Publishing will depend more on the availability of finances than on the quality of the work, distorting the concept of merit for careers, appointments and promotions.

This proves to be a typical case of the so-called Cobra Effect, which bedevils well-intended policies that fail to properly consider their unintended consequences.

### The cobra effect

The cobra effect loosely refers to unintended and unforeseen consequences of policies designed in good faith and with the view of bettering the current situation^25^. The term was originally introduced by Siebert^26^ to deride the unpredicted effects of poorly thought through financial incentives. It is based on a likely apocryphal anecdote about an attempt by the British Governor of colonial India to reduce the number of snakes roaming the street of New Delhi. He ruled that any citizen bringing to the city hall a dead cobra would get a cash reward. In no time the streets were cleared of snakes. However, people liked the relatively easy money, and began to breed cobras in their backyards, to then kill them to cash them in. The British authorities felt ridiculed, and abruptly stopped any reward for serpents’ carcasses. Indians did not know what to do with the cobras in their garden cages and freed them. The outcome was that there were many more cobras gliding through the streets of New Delhi than when the original rule had been introduced. The unforeseen consequences of OA resonate with the Cobra Effect.

### Open Access

Publishing in OA is on the increase. The lofty founding principles of OA were to counter the power and fight against the revenues of established, private publishing houses by making freely available all papers reporting studies funded by public money^27^. Initially, the idea was based both on the naïve concept that online publishing would not cost much, and that such costs could be sustained by international agencies sponsoring scientific publication world-wide like modern Mecenates.

However, there is no such a thing as a free lunch, and soon it became clear that the authors themselves had to fork out the expenses of OA, hence draining resources from the research process itself. Moreover, far from decreasing the market dominance of the established publishing companies, OA boosted their income by adding authors’ publishing fees to the subscriptions (the so-called “hybrid-journal” format), whilst increasing academic costs. The most harmful outcome of OA though has been paving the way to predatory publishing.

### Predatory publications

**Figure 1.**
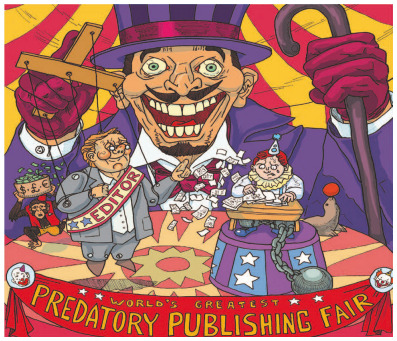
The cover illustration of *Cortex*, vol. 90, May 2017, drawn by Dario Battisti, depicting “The circus of predatory publishing.” Available from: https://www.sciencedirect.com/science/article/pii/S0010945217301090. Accessed on: Jan 18, 2022.

Predatory publishing is a pandemic that has infected science dissemination^28^. It is based on the OA model, whereby authors pay for the privilege of seeing their work in print, but, unlike the original OA vision, without the essential quality controls (Figure 1). These journals do not run proper peer-review processes, nor do they exert a sound editorial checking^29^. The model is very much like that of vanity press: pay-to-publish. Anything gets published, as hilariously demonstrated by the wonderfully goliardic article by two American scientists, who fed up with the constant email solicitations to submit their work to one or another such journals, eventually submitted a paper composed only of the sentence “Get me off your f***ing mailing list” repeated for 10 pages and illustrated by figures and graphs using the same text^30^. The roll-call of such imaginative hoaxes is long and ever increasing (see “List of scholarly publishing stings” in Wikipedia), proving beyond doubt that hundreds of journals operate below morally acceptable quality standards, and making a mockery of serious science.

Yet, blinded by their hubris^31^, or unashamed of taking shortcuts to boost their cv^32^, or allured by pecuniary gains^33^, researchers fall prey to or collude in these scams. They do not notice, or ignore, the flimsiness of the facades supporting these enterprises^34^, including fake impact factors^35^ and fake (or incompetent) editorial boards^36^. They are not deterred by the sloppy or non-existent vetting offered by these outlets. On the contrary, predatory publishers conquer larger and larger slices of the market, and paradoxically since these articles are OA, they end up being quoted even more than solid studies in non-OA journals^37^. The existence of such predatory outlets has also nurtured the phenomenon of paper mills, which offer shoddy, patchwork manuscripts for sale to unprincipled authors wishing to advance career effortlessly^38^.

The advent of these predatory outlets represents a real menace to the integrity of science dissemination^39^. The only way to dissuade scientists and academics from this immoral practice would be to make disadvantageous to publish in or edit for scam journals, which should count as a negative factor in appointments, advancements, awards and grant funding^28^. Authors should ignore papers appearing in predatory outlets^40^, even if those who published their work in such journals, unaware of the con, may feel some cognitive dissonance. However, countering their growth is challenging, not least because respectable publishing houses have launched many of their own OA spin-off journals, rendering the identification of predatory operations more ambiguous^41^.

The well-meant Plan S and the crooked predatory marked are two sides of the same coin: in a market dominated by pay-to-publish, who will have an interest in guaranteeing rigor and quality? Not publishing companies, who will gain more by publishing more, not the researchers who may jump at the chance of easy publication and not the readers who, not realising they may be exposed to drivel, will enjoy free access to journals previously hidden by paywalls.

Fortunately, not all is contemptible; there are also good OA initiatives, including journals managed by Learned Societies, as well as new formats promoting thorough science, like Pre-Registrations also available in OA regime.

### Pre-registration

To counter publication biases and poor methodology, the format of Registered Reports was nearly launched a decade ago. Registered Reports is a format of publication whereby the proposed experiments are peer reviewed before the research is carried out and, if accepted, will be published independently of the results. The format was first adopted by Cortex in 2012^42^ and, thanks to the unflinching determination of Chris Chambers, has spread to hundreds of other outlets^43^. This format guarantees quality and is less prone to the hurry imposed by quick and dirty reviewing style to accept all submissions for publication, as it aims at constructively assisting authors to better their study before embarking in data collection. Registered Reports offer a bulwark against the tide of substandard reports, at least until predatory outlets will annex this format as well.

The scale and severity of the problem is daunting. The scientific community should actively discourage the shortcuts of deceiving publishing, promoting a thoughtful and responsible dissemination, and embracing ethical reporting and sharing of data, putting an end to the current pandemic of unsound and immoral practices.
